# The imbalance between Tregs, Th17 cells and inflammatory cytokines among renal transplant recipients

**DOI:** 10.1186/s12865-015-0118-8

**Published:** 2015-09-23

**Authors:** Liang Ma, Huimao Zhang, Kebang Hu, Guoyue Lv, Yaowen Fu, Desalegn Admassu Ayana, Pingwei Zhao, Yanfang Jiang

**Affiliations:** Genetic Diagnosis Center, The First Hospital of Jilin University, Changchun, 130021 China; Department of Nephrology, The First Hospital of Jilin University, Changchun, 130021 China; Department of Gastroenterology, The First People’s Hospital of Changzhou, Third Affiliated Hospital of Suzhou University, Changzhou, Jiangsu 213003 China; Department of Medical Laboratory Sciences, Haramaya University, Haramaya, Ethiopia; Jiangsu Co-innovation Center for Prevention and Control of Important Animal Infectious Diseases and Zoonoses, Yangzhou, 225009 China

## Abstract

**Background:**

A significant barrier to organ transplantation is the cellular rejection that occurs and mediated by antibodies, T cells, and innate immune cells. This study was aimed to determine the number of CD4^+^CD25^+^Foxp3^+^ Treg, CD4^+^IFN-γ^−^IL-17^+^ Th17, CD4^+^IFN-γ^+^IL-17^−^ Th1 and CD4^+^IFN-γ^+^IL-17^+^ Th1/17 cells in renal transplant recipients (RTR).

**Methods:**

Renal transplantation was performed for a total of 35 patients with end-stage renal failure. The number of CD4^+^CD25^+^Foxp3^+^ Treg, CD4^+^IFN-γ^−^IL-17^+^ Th17, CD4^+^IFN-γ^+^IL-17^−^ Th1 and CD4^+^IFN-γ^+^IL-17^+^ Th1/17 cells, and the serum level of IFN-γ, TNF-α, IL-2, IL-4, IL-6, IL-10, and IL-17 were measured in pre- and post-transplant patients and 10 healthy controls (HC) using flow cytometry and Cytometric Bead Array (CBA). The association between the number of different subsets of CD4^+^ T-cells and clinical parameters were analyzed among the pre- and post-transplant patients, and the healthy controls.

**Results:**

The number of CD4^+^IFN-γ^−^IL-17^+^ Th17, CD4^+^IFN-γ^+^IL-17^−^ Th1 and CD4^+^IFN-γ^+^IL-17^+^ Th1/17 cells were significantly increased in patients with End-Stage Renal Failure (ESRF) compared to the HC. Stratification analysis indicated that AMR (Acute antibody mediated acute rejection), AR (acute rejection) and CR (chronic rejection) groups displayed greater number of CD4^+^IFN-γ^−^IL-17^+^ Th17, CD4^+^IFN-γ^+^IL-17^−^ Th1 and CD4^+^IFN-γ^+^IL-17^+^ Th1/17 cells as well as high level of serum IL-2, IFN-γ, TNF-α and IL-17. But, the AMR, AR and CR groups have shown lower level of CD4^+^CD25^+^Foxp3^+^ T cells and serum IL-10 compared to transplant stable (TS) patients. Moreover, the number of Tregs were negatively correlated with the number of Th17 cells in RTR patients. The number of Tregs and Th17 cells were positively correlated with the eGFR and serum creatinine values, respectively.

**Conclusion:**

The imbalance between different types of CD4^+^ T cells and dysregulated inflammatory cytokines may contribute towards renal transplantation rejection.

## Background

Renal transplantation is used to improve survival and quality of life for patients with end-stage renal disease. In the past, patients often eventually die from complications [1, 2] if toxins cannot be removed from the body by hemodialysis. Although renal transplantation is recognized as the gold strategy for treating renal failure, it has several limitations including donor’s immune rejection. In order to identify a means of controlling immune rejection, further illustration on the mechanism of immune rejection in renal transplant recipients (RTR) has great significance.

It is generally accepted that a significant barrier to organ transplantation is the humoral and cellular rejection that can occur and mediated by antibodies, T cells, and innate immune cells. Cellular immune response play’s an equally important role with humoral immune response in allograft rejection [3, 4]. For instance, there is evidence that a disturbed T-cell homeostasis play’s a critical role in the development of acute graft rejection episodes. The main T subsets which are pivotal for this T-cell balance consists of T-helper 17 (Th17) cells and regulatory T (Treg) cells [5–7]. In addition to well characterized Th1 and Th2 lymphocytes, additional subsets called Th17 cells, which selectively produce IL-17, have joined the effector CD4^+^ T cell lineage. Imbalanced Th17 and impaired Treg cells have suggested to be involved in the pathogenesis of allograft rejection, such as heart and lung transplantations [8–11]. Previous studies have suggested that Th17 cells are important for clearance of a variety of pathogens and are associated with numerous autoimmune and inflammatory conditions [12]. In addition, Th17 cells have also been implicated in acute and chronic rejection in animal models of allograft transplant [13–16]. Interestingly, the function of self-reacting effector Th17 cells is controlled by Tregs, yet another subpopulation of CD4^+^ T lymphocytes which express transcription factor FoxP3 [17]. Tregs are important regulators of immune tolerance and can actively suppress pro-inflammatory T cell responses [18, 19]. Quantitative and/or qualitative deficiencies of Tregs have been associated with the development of organ transplantation rejection [20–23]. Previous studies in animal models have shown that a deficiency in Tregs favors kidney transplantation rejection [20, 21], though their mechanism in clinical studies remains unclear. Human Tregs are not as well characterized as their murine counterparts; in part this is due to restrictions and limitations of clinical studies. Furthermore, the characterization of Tregs in humans is more complex [24, 25]. Human Tregs are CD4^+^CD25^+^ and their development and function depends on the forkhead family transcription factor (Foxp3) expression [26–28]. Recent study has shown that a lower frequency of circulating CD4^+^CD25^+^Foxp3^+^ T cells was detected in RTR patients, and the percentages of CD4^+^CD25^+^Foxp3^+^ T cells were negatively associated with eGFR of RTR [29]. However, little is known about the number of Tregs and Th17 cells, and their association with different types of rejection in RTR patients.

In addition, studies have shown that some inflammatory cytokines, such as Th1-type cytokine (IFN-γ) and Th17-type cytokine (IL-17), are also associated with the development of rejection [30–33]. For instance, IFN-γ can mediate separate functions at the same target organ during Graft-versus-host disease (GVHD), and IL-17 can induce the expression of proinflammatory tumor necrosis factor (TNF)-α, chemotactic protein (MCP)-1 and macrophage inflammatory protein (MIP)-1 to promote tissue inflammation [30–32]. Furthermore, IL-17 can also promote the differentiation and maturation of dendritic precursor cells, increased cell surface expression of CD80, CD40 and major histocompatibility complex (MHC)-II antigen [33]. However, the role of these inflammatory cytokines in different types of renal transplantation rejection has not been clarified.

In the current study, we characterized the number of circulating CD4^+^CD25^+^Foxp3^+^ Tregs and Th17 cells, and the concentration of serum inflammatory cytokines in different RTR patients and HCs to determine their potential association with clinical measures in the patients.

## Methods

### Subjects

A total of 35 patients (20 male and 15 female), 31–48 years of age (median age 38), with an end-stage renal failure (ESRF) waiting for renal transplantation were recruited from the inpatient service of the First Hospital of Jilin University, Changchun, China. Ten gender, ethnicity and age-matched HCs were included in the study. Individual patients with ESRF were diagnosed according to the criteria [34]. Individual patients were treated with conventional immunosuppressors (Cyclosporine, azathioprine or mycophenolate mofetil and/or steroids) twice a day for three days. All patients had compatible HLA-gene matches and the number of those patients who had HLA (A, B, DR) mismatches were less than two. Individuals were excluded if she/he had a history of previous renal transplantation or surgical procedure. Written informed consent was obtained from individual participants. The experimental protocol was established according to the guidelines of the 1975 Declaration of Helsinki and approved by the Human Ethics Committee of Jilin University, China.

### Study groups

Transplant patients were divided into four groups according to graft function (based on estimated glomerular filtration rate (eGFR) or serum creatinine level) and post-transplant rejection time (after 12 weeks) as well as the Banff Classification [35]. The four groups included were: (a) Transplant Stable (TS) (*n* = 13): Recipients with stable graft function under conventional immunosuppressors (Cyclosporine, azathioprine or mycophenolate mofetil and/or steroids) and without clinical and laboratory features suggestive of rejection (serum creatinine level: < 150 umol/L; eGFR: ≥ 50) in 12 weeks; There was no biopsies of these individuals because they have normal and stable graft function; (b) Acute antibody mediated acute rejection (AMR) (*n* = 8): Recipients under conventional immunosuppressors with clinical symptoms (fever, graft pain, oliguria or anuria) and progressive renal function deterioration (serum creatinine level: ≥ 150 umol/L; eGFR: < 50) during 1 week; The rejection of these individuals was confirmed by biopsy according to Banff criteria; (c) Acute rejection (AR) (*n* = 7): Recipients under conventional immunosuppressors with clinical symptoms (fever, graft pain, oliguria or anuria) and progressive renal function deterioration (serum creatinine level: ≥ 150 umol/L; eGFR: < 50) during 2 to 12 weeks; The rejection of these individuals was confirmed by biopsy according to Banff criteria; (d) Chronic rejection (CR) (*n* = 7): Recipients under conventional immunosuppressors with clinical symptoms (fever, graft pain, oliguria or anuria) and progressive renal function deterioration (serum creatinine level: ≥ 150 umol/L; eGFR: < 50) after 12 weeks; The rejection of these individuals was confirmed by biopsy according to Banff criteria. These group of patients who received kidney transplant had no post-transplant malignancy and infective complications. In case of rejection response, patients withdrew from the follow-up and receive additional anti-rejection treatment, such as methyl prednisolone, anti-thymocyte globulin or anti-CD3 monoclonal antibody as well as plasma exchange.

### Clinical measurement

Peripheral venous blood samples were obtained from individual participants for laboratory test before and after transplant, when rejection-related clinical symptoms appeared. The routine laboratory investigations include complete blood count, serum creatinine, BUN and glomerular filtration rate (eGFR). The laboratory investigations were conducted by scattered turbidimetry using Siemens special protein analyzer (Siemens Healthcare Diagnostics Products, GmbH, Germany).

### Isolation and stimulation of PBMCs

Peripheral venous blood samples were collected after overnight fasting. Peripheral blood mononuclear cells (PBMCs) were isolated by density-gradient centrifugation using Ficoll-Paque Plus (Amersham Biosciences, Little Chalfont, UK). PBMCs (10^6^/mL) were stimulated in duplicate with 50 ng/mL of phorbol myristate acetate (PMA) and 1.0 mg/mL of ionomycin (Sigma, St. Louis, MO, USA) in 10 % human AB type sera in RPMI 1640 medium at room temperature in a humidified incubator with 95 % air and 5 % carbon dioxide for 2 h and then cultured for another 4 h in the presence of 0.5 mg/mL of brefeldin A (BFA, Sigma). The control PBMCs were cultured in medium alone.

### Flow cytometry analysis

The frequency of CD4^+^IFN-γ^+^IL-17^−^ Th1, CD4^+^IFN-γ^−^IL-17^+^ Th17, CD4^+^IFN-γ^+^IL-17^+^ Th1/17 and CD4^+^CD25^+^Foxp3^+^ T cells in individual samples were determined by flow cytometry following intracellular staining with anti-cytokine antibodies. Briefly, the stimulated PBMCs were harvested and stained with allophycocyanin (APC)-labeled anti-CD4, fixed with the Perm/Fix solution, and permeabilized, followed by staining with fluorescein isothiocyanate (FITC)-labeled anti–IL-17 and PE-Cy7-labeled anti-IFN-γ (Becton Dickinson, San Diego, USA). Additional cells were stained in duplicate with PerCP-anti-CD4/FITC-anti-CD25 or isotype-matched controls (BD PharMingen, San Diego, USA) for 30 min, fixed, and permeabilized using the permeabilization solution (BD Biosciences), followed by intracellular staining with PE-anti-Poxp-3 (BD PharMingen, San Diego, USA). After being washed with PBS, these cells were analyzed on a FACSCalibur (BD Biosciences, San Jose, USA) and at least 20,000 events were analyzed by FlowJo software (v7.6.2).

### Cytometric bead array for the level of serum cytokines

The concentrations of serum IFN-γ, TNF-α, IL-2, IL-4, IL-6, IL-10 and IL-17 were determined by cytometric bead array (CBA), according to the manufacturer’s protocol (CBA, BD Biosciences) with minor modification. Briefly, 50 μL serum samples were subjected to analysis in duplicate using the cytometric bead array kit on a FACSCalibur cytometry. The concentration of serum cytokines were quantified using CellQuestPro and CBA software (Becton Dickinson). The detection limit for IFN-γ, TNF-α, IL-2, IL-4, IL-6, IL-10 and IL-17 were 4.1 pg/ml, 3.7 pg/ml, 2.9 pg/ml, 3.3 pg/ml, 2.5 pg/ml, 3.3 pg/ml, and 4.2 pg/ml, respectively.

### Statistical analysis

Data were expressed as median and range for each group unless specified. The difference between groups was analyzed by the Kruskal-Wallis test or Chi-square test using SPSS 16.0 software for unpaired and paired comparisons, respectively. The relationship between variables was evaluated using the Spearman rank correlation test. A two-side *P* value < 0.05 was considered statistically significant.

## Results

### Patient sociodemographic and clinical characteristics

The sociodemographic and clinical characteristics of RTR’s are summarized in Table 1. The patients displayed higher concentration of serum creatinine and BUN and lower level of eGFR. Furthermore, significantly higher levels of white blood cell (WBC), and lower levels of red blood cell (RBC) and hemoglobin (Hb) were detected in ESRF, compared to the HCs.

**Table 1:** The demographic and clinical characteristics of subjects

Parameters	HC	ESRF
Number	10	35
Age (years)	37 (30–45)	38 (31–48)
F/M (n)	4/6	15/20
BUN (mmol/L)	4.4 (3.5–7.0)	16.8 (7.2–32.8) *
Cr (umol/L)	88 (65–112)	832 (499–1790) *
eGFR (ml/min)	115 (98–124)	21 (8–67) *
WBC (×10^9^/L)	7.7 (3.6–9.4)	10.9 (8.5–13.9)*
PBMCs (×10^9^/L)	2.7 (0.9–4.5)	2.1 (1.1–3.3) *
RBC (×10^12^/L)	4.0 (3.7–4.6)	2.4 (1.7–3.1)
Hb (g/L)	128 (111–147)	85 (66–107)

Data shown are median (range) of each group of subjects

*ESRF* End-stage renal failure, *BUN* Blood urea nitrogen (normal range: 3.2 ~ 6.0 mmol/L), *Cr* Serum creatinine (normal ranges: men: 44-133umol/L; women: 70-108umol/L), *eGFR* Glomerular filtration rate (normal value: 125 ml/min), *WBC* White blood cell (normal range: 4 ~ 10 × 10^9^/L), *RBC*: Normal range: men, 4.0 ~ 5.5 × 10^12^/L; women, 3.5 ~ 5.0 × 10^12^/L; Hb: normal range: men, 120-160 g/L; women, 110–150 g/L

*P < 0.05 vs. The HC

RTR’s were divided into four groups according to graft function (based on eGFR or serum creatinine level) and post-transplant rejection time (Table 2). TS Patients showed a significant reduction in BUN and Cr levels and a significant increase in eGFR values and Hb compared to the pre transplant status. In addition, the concentration of serum Cr and BUN in AMR, AR and CR groups were significantly higher than TS ESRF patients.

**Table 2:** Clinical characteristics of before and post-transplant patients

	Transplant stable (*n* = 13)		Acute antibody mediated acute rejection (*n* = 8)		Acute rejection (*n* = 7)		Chronic rejection (*n* = 7)	
	Before	After	Before	After	Before	After	Before	After
BUN (mmol/L)	17.5 (7.2–32.8)	7.4* (3.7–10.6)	16.2 (10.1–28.4)	29.1* (22.3–38.9)	16.2 (14.4–20.6)	25.2* (17.9–34.7)	16.5 (9.2–22.6)	18.3* (13.5–26.8)
Cr (umol/L)	779 (499–1790)	130* (72–352)	798 (739–1682)	1387* (1098–1786)	789 (762–1562)	987* (794–1682)	786 (698–1492)	578* (512–755)
eGFR (ml/min)	20 (8–67)	88* (59–101)	27 (8–62)	9* (4–13)	21 (9–59)	12* (6–24)	24 (9–51)	18* (5–35)
WBC (×10^9^/L)	9.8 (8.5–13.4)	7.9* (6.3–9.7)	9.7 (8.9–13.9)	17.7* (11.8–19.6)	9.6 (8.8–11.6)	13.1* (9.9–16.2)	9.7 (8.6–13.2)	11.7* (9.6–13.7)
PBMCs (×10^9^/L)	1.7 (1.1–2.9)	0.8* (0.6–1.0)	1.7 (1.3–3.3)	0.3* (0.1–0.6)	1.9 (1.4–3.1)	0.7* (0.4–1.0)	1.7 (1.1–2.7)	0.7* (0.9–1.2)
RBC (×10^12^/L)	2.4 (2.1–2.7)	3.1* (2.6–3.3)	2.0 (1.7–2.5)	1.5* (1.3–2.0)	2.1 (1.9–3.1)	1.9* (1.7–2.3)	2.0 (1.9–2.9)	2.3* (2.0–2.8)
Hb (g/L)	86.0 (73.0–99.0)	97.0* (88.0–103.0)	82.0 (66.0–91.0)	73.0* (61.0–77.0)	87.0 (69.0–103.0)	78.0* (67.0–89.0)	84.0 (69.0–107)	91.0* (80.0–110.0)

Data are expressed as median (range) or real case numbers

* *P* < 0.05 vs, before transplant rejection

### CD4^+^IFN-γ^−^IL-17^+^ Th17, CD4^+^IFN-γ^+^IL-17^−^ Th1 and CD4^+^IFN-γ^+^IL-17^+^ Th1/17 T cells in the patients

**Fig. 1:**
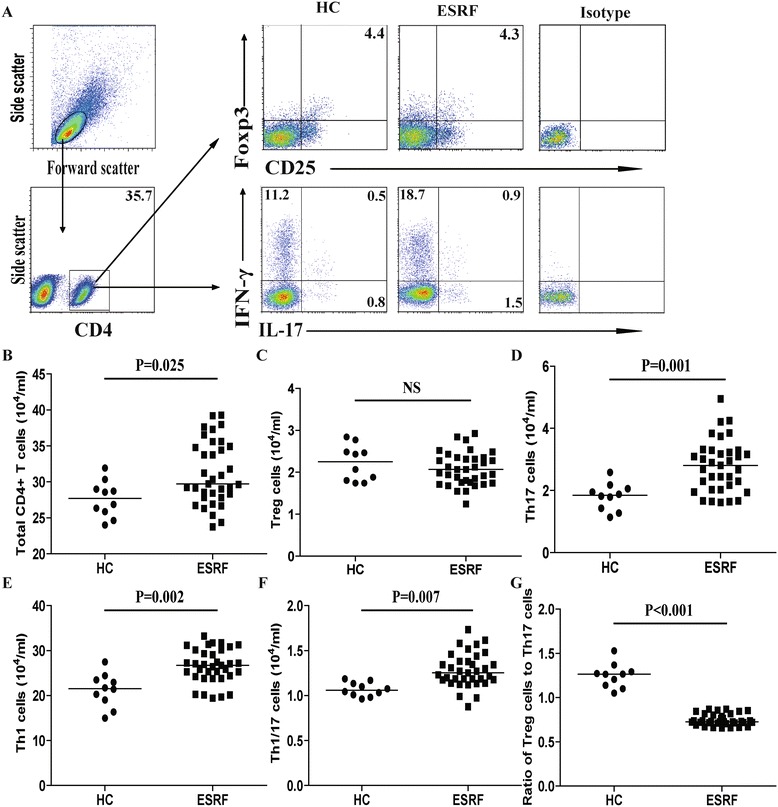
FACS analysis of circulating CD4^+^ T cells. PBMCs from individual ESRF patients and HC subjects were stained with APC-anti-CD4, or PerCP-anti-CD4 and FITC-anti-CD25, or isotype controls, fixed and permeabilized, followed by intracellular staining with FITC-anti–IL-17 and PE-Cy7-anti-IFN-γ and PE-anti-Foxp3. The frequency of CD4^+^, CD4^+^CD25^+^Foxp3^+^ Treg, CD4^+^IFN-γ^−^IL-17^+^ Th17, CD4^+^IFN-γ^+^IL-17^−^ Th1 and CD4^+^IFN-γ^+^IL-17^+^ Th1/17 cells were determined by flow cytometry analysis. The cells were gated on living lymphocytes and then gated on CD4^+^ cells, and at least about 30,000 events were analyzed for each sample. The numbers of each type of CD4^+^ T cells were calculated, according to the total numbers of PBMCs, the frequency of total CD4^+^, and different types of CD4^+^ T cells. **a** The representative charts of flow cytometry analysis. **b-g** Quantitative analysis. Data shown are representative FACS charts or the mean numbers of each type of cells per ml of peripheral blood in individual subjects from two separate experiments. The horizontal lines indicate the median values for each group

The circulating CD4^+^ T cells analysis (Fig. 1a-g) found that, the number of CD4^+^, CD4^+^IFN-γ^−^IL-17^+^ Th17, CD4^+^IFN-γ^+^IL-17^−^ Th1 and CD4^+^IFN-γ^+^IL-17^+^ Th1/17 cells (Fig. 1a, d-f) in patients with ESRF were significantly greater than the HCs. In contrast, there was no significant difference in the number of CD4^+^CD25^+^Foxp3^+^ T cells (Fig. 1b) between the ESRF patients and HCs. As a result, the ratio of CD4^+^CD25^+^Foxp3^+^ T cells to CD4^+^IFN-γ^−^IL-17^+^ Th17 cells (Fig. 1g) was significantly lower in ESRF patients compared to the HCs. Furthermore, there was no significant difference in the ratio of CD4^+^CD25^+^Foxp3^+^ T cells to CD4^+^IFN-γ^+^IL-17^−^ Th1 or CD4^+^IFN-γ^+^IL-17^+^ Th1/17 cells between ESRF patients and the HCs.

### The number of CD4 + IFN-γ-IL-17+ Th17, CD4 + IFN-γ + IL-17- Th1, CD4 + IFN-γ + IL-17+ Th1/17 T and CD4 + CD25 + Foxp3+ T cells

**Fig. 2:**
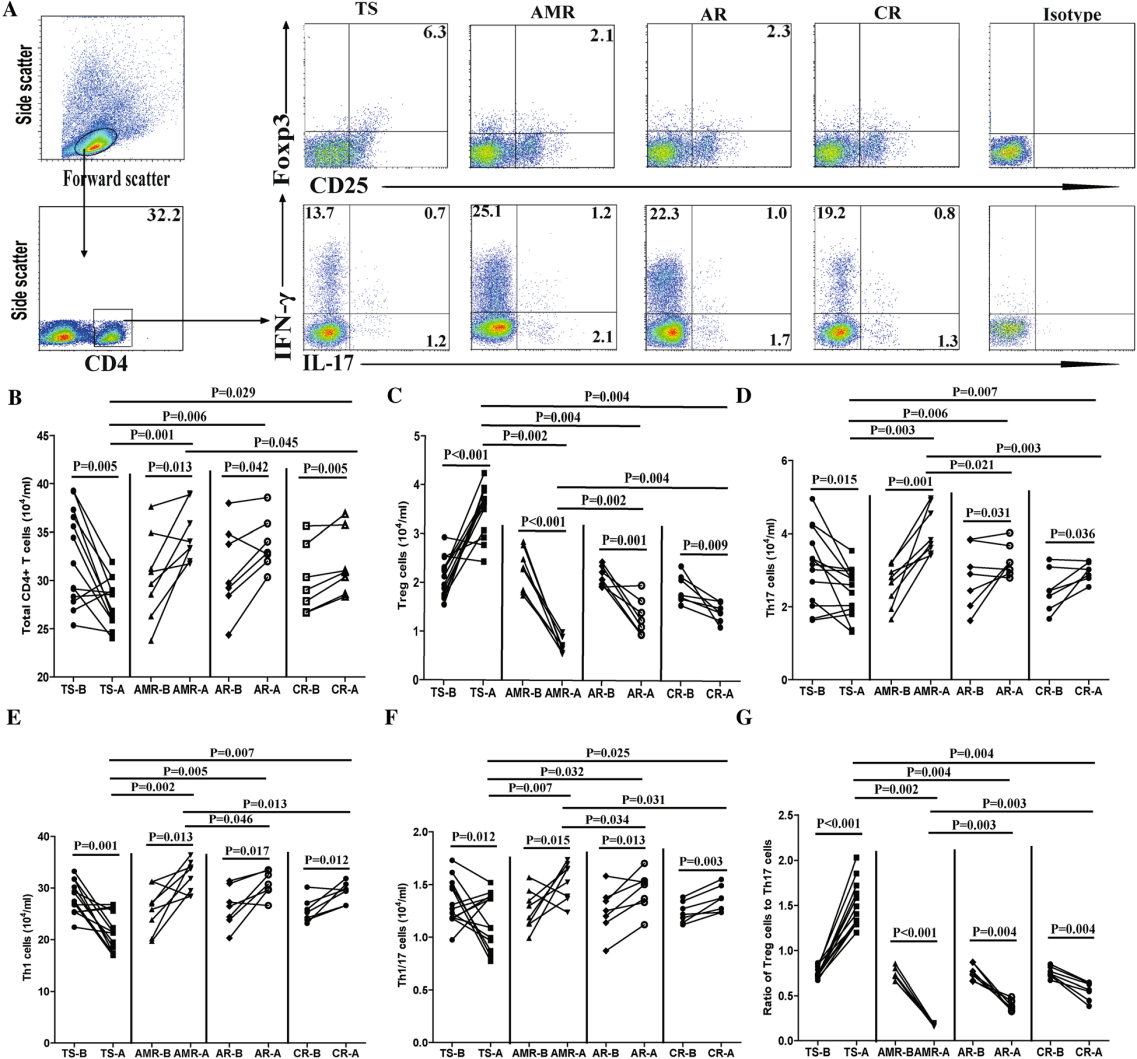
Stratification analysis of the numbers of different types of CD4^+^ T cells in different groups of renal transplant patients. The patients were stratified into the transplant stable (TS) (13 cases), accelerated rejection (AMR) (8 cases), sub-acute rejection (AR) (7 cases) and chronic rejection (CR) (7 cases) groups, the numbers of CD4^+^, CD4^+^CD25^+^Foxp3^+^ Treg, CD4^+^IFN-γ^−^IL-17^+^ Th17, CD4^+^IFN-γ^+^IL-17^−^ Th1 and CD4^+^IFN-γ^+^IL-17^+^ Th1/17 cells and the ratios of Tregs to Th17 cells in these four groups of patients were analyzed. **a** The representative charts of flow cytometry analysis. **b-g** Quantitative analysis. Data shown are representative FACS charts or the mean numbers of each type of cells per ml of peripheral blood in individual subjects from two separate experiments. The horizontal lines indicate the median values for each group

Further comparison of different types of CD4^+^ T cells (Fig. 2a-g) found that, in post-transplant TS patients, the number of CD4^+^CD25^+^Foxp3^+^ T cells and the ratio of Tregs to Th17 were significantly increased (Fig. 2c, g), whereas the number of CD4^+^, CD4^+^IFN-γ^−^IL-17^+^ Th17, CD4^+^IFN-γ^+^IL-17^−^ Th1 and CD4^+^IFN-γ^+^IL-17^+^ Th1/17 cells were significantly decreased compared to the pre-transplant status. (Fig. 2b, d-f). Conversely, in the AMR, AR and CR patients, the number of CD4^+^CD25^+^Foxp3^+^ T cells is decreased while the numbers of CD4^+^IFN-γ^−^IL-17^+^ Th17, CD4^+^IFN-γ^+^IL-17^−^ Th1 and CD4^+^IFN-γ^+^IL-17^+^ Th1/17 cells are increased (Fig. 2b-g). Moreover, there was no significant change in the ratio of Tregs to Th1 cells in the pre- and post-transplant status. Interestingly, AMR patients displayed higher number of CD4^+^, CD4^+^IFN-γ^−^IL-17^+^ Th17, CD4^+^IFN-γ^+^IL-17^−^ Th1 and CD4^+^IFN-γ^+^IL-17^+^ Th1/17 cells and lower number of CD4^+^CD25^+^Foxp3^+^ T cells compared to AR and CR patients. These data suggested that altered CD4 ^+^ T cells number may be associated with renal transplantation rejection.

### Serum Th1- and Th17-type cytokines in RTR patients

**Fig. 3:**
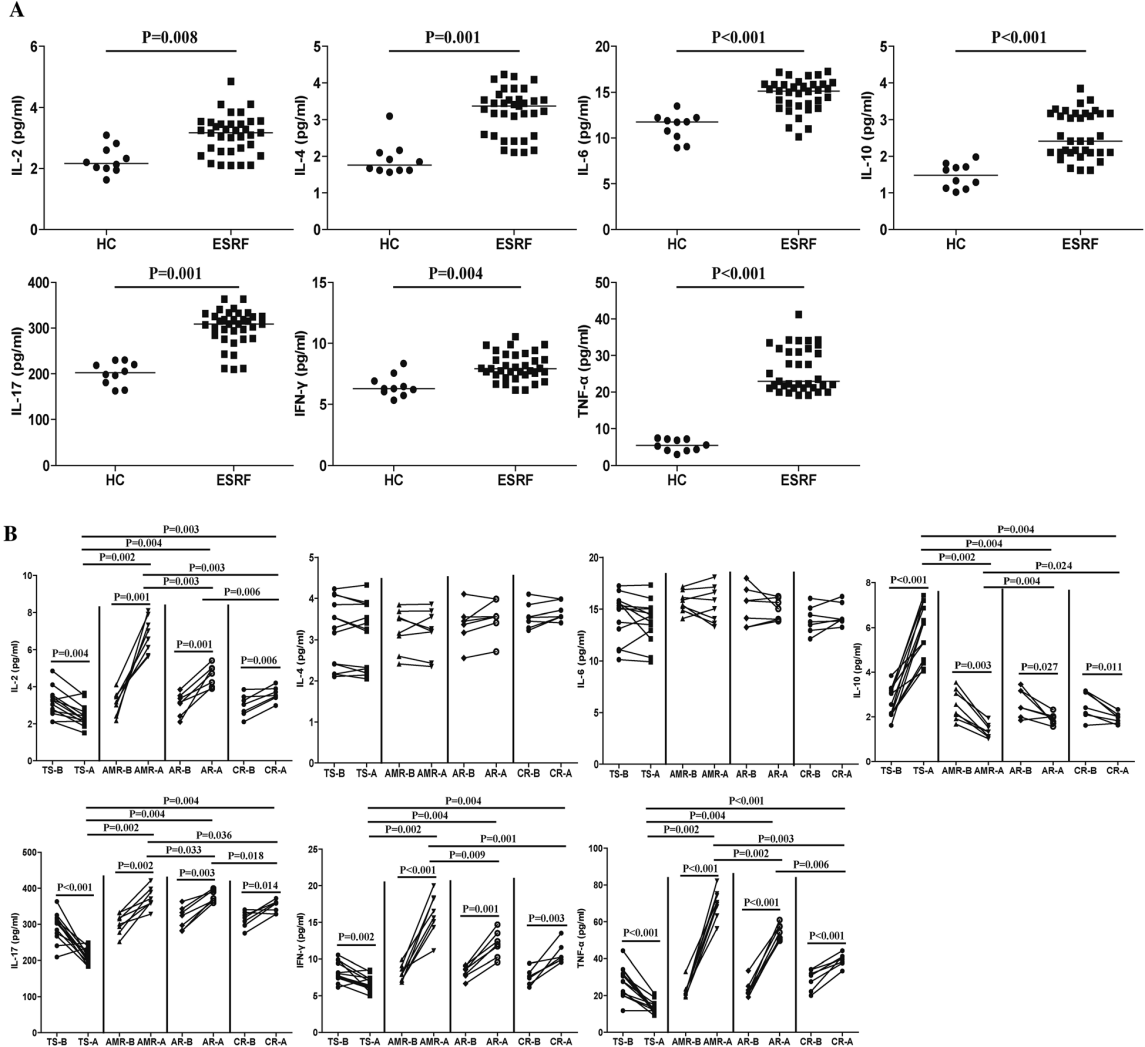
The levels of serum inflammatory cytokines in ESRF and RTR patients. **a** The levels of serum IFN-γ, TNF-α, IL-2, IL-4, IL-6, IL-10 and IL-17 in HCs and ESRF patients. **b** Plasma levels of serum IFN-γ, TNF-α, IL-2, IL-4, IL-6, IL-10 and IL-17 in patients at before and post-transplant in these groups of patients were analyzed. The horizontal lines indicate the median values of the different groups

The concentration of serum IL-2, IFN-γ, TNF-α, IL-4, IL-6, IL-10 and IL-17 in ESRF patients were significantly increased compared to the HC’s (Fig. 3a). The serum cytokines level were determined and found significantly increased serum levels of IL-2, IFN-γ, TNF-α and IL-17 in post-transplant TS’s compared to the pre-transplant patients. TS patients showed an increased level of serum IL-10 compared to the pre-transplant RTR’s (Fig. 3b). However, there was no significant difference in the concentrations of IL-4 and IL-6 pre and post-transplant. These data indicated that higher serum levels of Th17- and Th1-type cytokines may be associated with renal transplantation rejection.

### The correlation of Tregs with Th17 cells in RTR patients

**Fig. 4:**
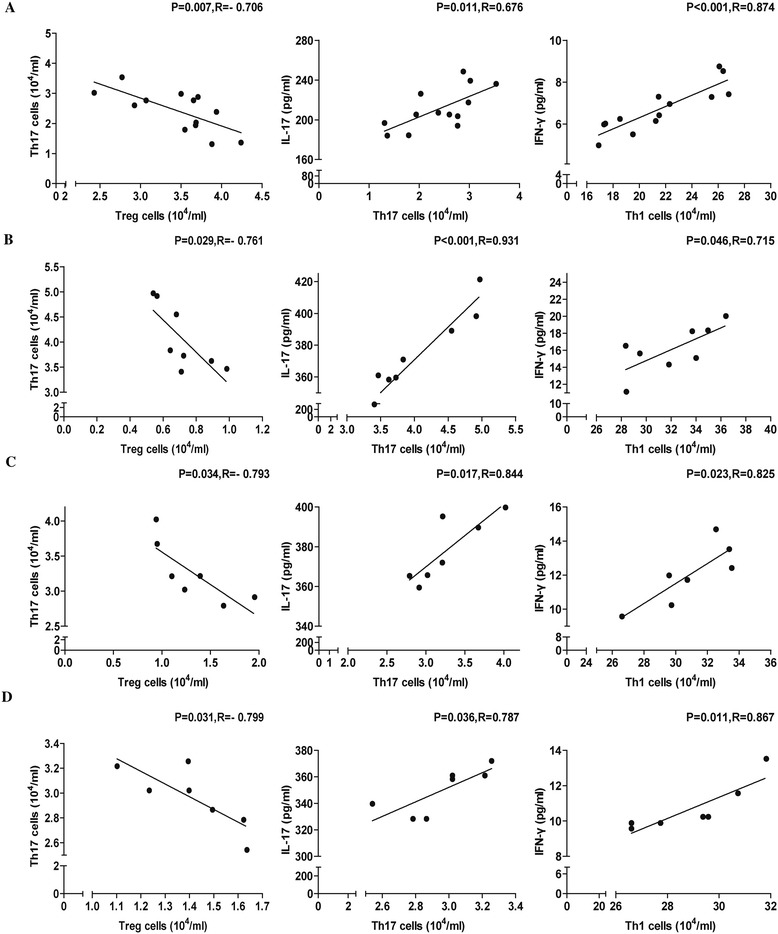
Correlation between the numbers of circulating CD4^+^ T cells and serum levels of inflammatory cytokines in RTR patients. Correlation between the numbers of CD4^+^CD25^+^Foxp3^+^ T, CD4^+^IFN-γ^−^IL-17^+^ Th17 and CD4^+^IFN-γ^+^IL-17^−^ Th1 cells and CD4^+^IFN-γ^−^IL-17^+^ Th17 cells, serum levels of IL-17 and IFN-γ in TS (**a**), AMR (**b**), AR (**c**) and CR (**d**) patients

The potential relationship between the concentration of serum inflammatory cytokines and the number of different subsets of CD4^+^ T cells in RTR patients was analyzed (Fig. 4a-d). It was found that, the concentration of serum IL-17 and IFN-γ was positively correlated with the number of CD4^+^IFN-γ^−^IL-17^+^ Th17 and CD4^+^IFN-γ^+^IL-17^−^ Th1 cells, respectively, in the four groups. Further analysis of the relationship found a negative association between the number of CD4^+^CD25^+^Foxp3^+^ T cells and the number of CD4^+^IFN-γ^−^IL-17^+^ Th17 cells in the four groups. These data suggested that different types of CD4^+^ T cells may have variable functions during renal transplantation rejection.

### The relationship between the number of CD4^+^ T cells or inflammatory cytokines and clinical parameters in TS patients

**Fig. 5:**
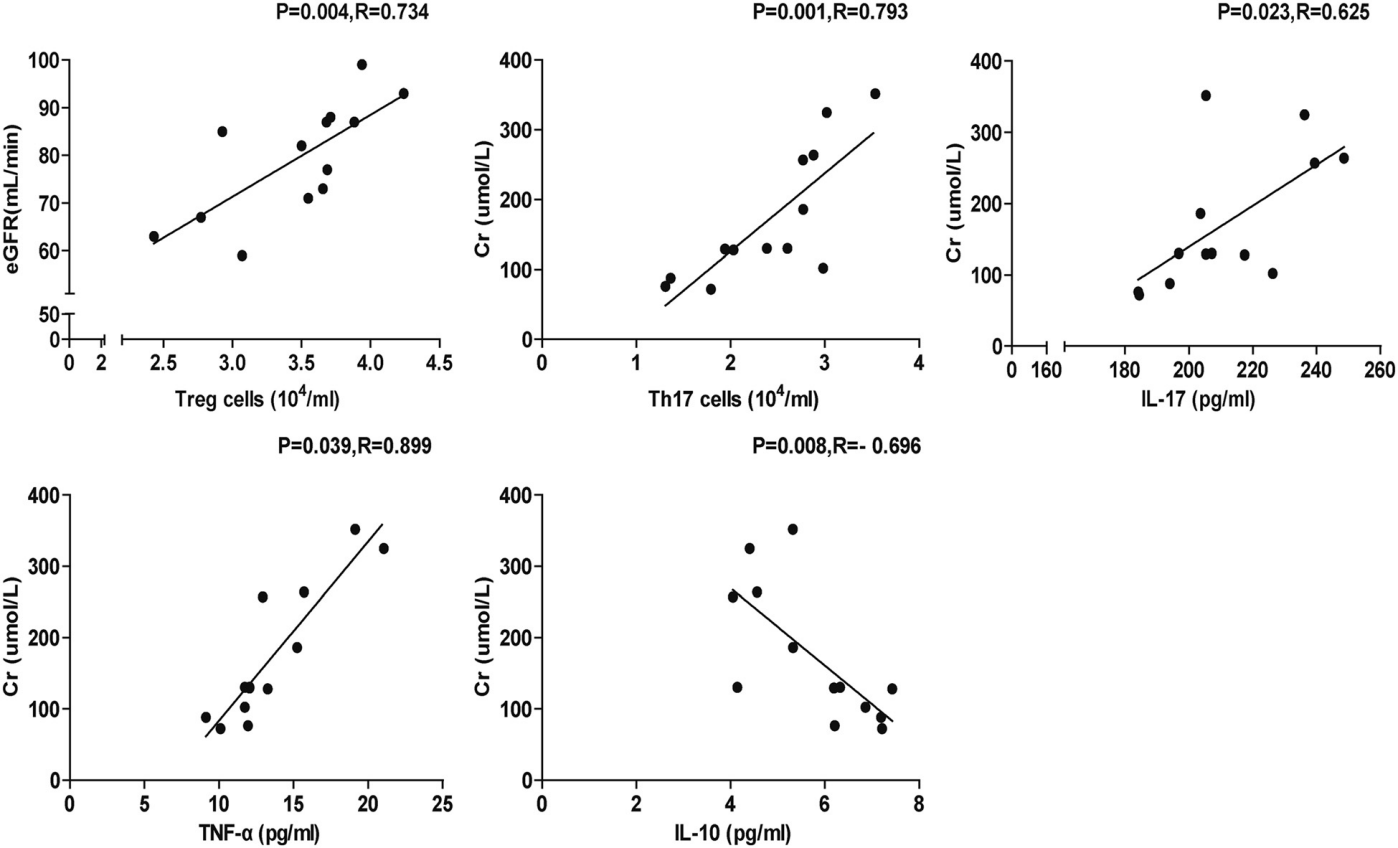
Correlation between the numbers of circulating CD4^+^ T cells or serum levels of inflammatory cytokines and disease activity in RTR patients. Correlation between the numbers of CD4^+^CD25^+^Foxp3^+^ T, CD4^+^IFN-γ^−^IL-17^+^ Th17 cells and serum levels of TNF-α, IL-17 and IL-10 and the valves of eGFR and the serum levels of Cr in TS patients

To understand the importance of different subsets of CD4^+^ T cells and inflammatory cytokines, we analyzed the potential association of the number of different types of CD4^+^ T cells and inflammatory cytokines with clinical parameters of the patients. It was found that the number of CD4 + CD25 + Foxp3+ and CD4 + IFN-γ-IL-17+ Th17 cells were positively correlated with the eGFR value and serum creatinine level, respectively, in the TS group (Fig. 5). Further analysis revealed that the serum level of TNF-α and IL-17 were positively correlated with serum creatinine level. In contrast, the serum level of IL-10 was negatively correlated with serum creatinine in the TS group. Moreover, three groups of RTR also showed the same trend (data not shown).

## Discussion

Although renal transplantation provides a readily accessible alternative strategy for patients with end-stage renal failure, immune rejection remains a major hurdle to its implementation. In this study, we examined the pre and post-transplantation number of different types of circulating CD4^+^ T cells in ESRF patients. The study found that, the number of CD4^+^IFN-γ^−^IL-17^+^ Th17, CD4^+^IFN-γ^+^IL-17^−^ Th1 and CD4^+^IFN-γ^+^IL-17^+^ Th1/17 cells in ESRF patients were significantly increased compared to the HC, whereas there was no significant difference in the number of CD4^+^CD25^+^Foxp3^+^ T cells between patients and HC’s. Apparently, there was an imbalance of Tregs to Th17 cells in ESRF patients. Together these data suggested that dysregulated CD4^+^ T cells play an important role in ESRF patients, which were consistent with previous studies [36]. Moreover, RTR showed increased number of CD4^+^IFN-γ^−^IL-17^+^ Th17, CD4^+^IFN-γ^+^IL-17^−^ Th1 and CD4^+^IFN-γ^+^IL-17^+^ Th1/17 cells and higher levels of serum IL-2, IFN-γ, TNF-α and IL-17, but decreased number of CD4^+^CD25^+^Foxp3^+^ T cells and lower levels of serum IL-10 compared to pre-transplant and TS’s patients. Conceivably, an imbalance in CD4^+^ T cells and inflammatory cytokines were new target for immunotherapy for the intervention of renal transplantation.

Increasing evidences have shown that multiple subsets of effector CD4^+^ T cells play an important role in allograft rejection [37, 38]. In addition to direct killing activity by cytotoxic T lymphocytes, organ allotransplantation rejection can occur through T-cell-mediated mechanisms, including cytokine production, recruitment and activation of other cytotoxic cells as well as B cells that produce xenoreactive antibodies [39]. This study demonstrated that after renal transplant, patients showed increased number of CD4^+^IFN-γ^+^IL-17^−^ Th1 cells and Th1-type cytokines (IL-2, IFN-γ, TNF-α) compared to the pre-transplant and TS patients, which was consistent with previous reports [40–42]. Similarly, we detected significantly higher levels of cytokines in post-transplant patients compared to pre-transplant. Moreover, the number of CD4^+^IFN-γ^+^IL-17^−^ Th1 cells was positively correlated with the concentration of serum IFN-γ in all the four post-transplant groups. However, there was no significant difference in the Th2-type cytokines (IL-4, IL-6) before and after transplant. These data suggested that Th1, not Th2, likely play an important role in rejection, which were consistent with Yuxin and coworkers [43]. The significantly changed Th1 cells may stem from the inflammatory environment, which preferably activate naive helper T cells towards Th1 direction. In addition to Th1 cells, Th17 cells expressing retinoic acid-related orphan receptor γt (RORγt) that play a crucial role in the development of transplant rejection by producing pro-inflammatory cytokine IL-17 [5–7]. Some studies have demonstrated that Th17 and IL-17 contribute to rejection during heart, lung, liver and other organ allotransplantation [8, 9]. Morever, similar studies have also suggested that IL-17-secreting cell infiltration is a prognostic marker for determining allograft outcome in renal allograft biopsies with acute T-cell-mediated rejection (ATCMR) [44, 45]. However, the possible mechanisms of Th17 and IL-17 in different types of renal transplantation rejection remains unknown. Notably, we detected significantly increased number of CD4^+^IFN-γ^−^IL-17^+^ Th17 cells and Th17-type cytokines (IL-17) in RTR compared to the pre-transplant status and TS patients. Moreover, the number of Th17 cells was positively correlated with serum IL-17 concentration in the post-transplant groups. These results suggested that Th17 cells, as well as Th1 cells, play a key role in renal transplantation rejection. More interestingly, the study found greater number of CD4^+^IFN-γ^+^IL-17^+^ Th1/17 cells in RTR group compared to pre-transplant and post-transplant TS patients. Previous studies have shown that CD4^+^IFN-γ^−^IL-17^+^ Th17 cells are a rare population and the development of these cells depends on the cytokine environment [46–48]. However, there was no significant correlation between the number of Th1/17 cells and the clinical parameters. It is possible that Th1/17 cells may not be potent effectors for transplant rejection. Given that, following activation, T cells can differentiate into different functional T cells, these Th1/17 cells may be early differentiated and uncommitted cells. We are interested to further investigating the role of Th1/17 cells in the renal transplantation rejection.

Similar studies have reported that CD4^+^CD25^+^Foxp3^+^ T cells play an important role in immune tolerance mechanism during renal transplantation rejection [22–24]. A study by Wen et al. [29] found that following transplantation, RTR had significantly lower levels of CD4^+^CD25^+^Foxp3^+^ T cells compared to ESRF and these cells had a positive linear relationship with glomerular filtration rate. This finding was consistent with our data that showed RTR had lower number of CD4^+^CD25^+^Foxp3^+^ T cells compared to pre-transplant status. This observation may partly reflect the impact of immunosuppression on this cell population. Studies of Tregs in RTR have shown variably changing circulating levels during post-transplantation, suggesting the possibility of regulatory cells production expressing Foxp3 in immunosuppressed RTR’s.

Further studies on CD4^+^ T-cell subsets in RTR have indicated that the number of CD4^+^CD25^+^Foxp3^+^ T cells were negatively correlated with the number of CD4^+^IFN-γ^−^IL-17^+^ Th17 cells, which was supported by molecular studies showing the presence of reciprocal interaction between these subpopulation. Though, both Th17 and Tregs require TGF-β1 during the early stage of differentiation, in the presence of pro-inflammatory cytokines TGF-β1 and IL-6, FoxP3 is down-regulated and T-cell with transitional phenotype express a set of proteins essential for Th17 development [49]. Additionally, IL-2, which is required for the regulation of Foxp3 expressing Tregs, has been found to inhibit the development of Th17 cells [50]. Hence, it is possible that the counteractive effects of IL-2 and IL-6 on the differentiation of Th17 and Treg in the periphery may hamper immunoregulatory responses and facilitate the persistence of rejection. Morever, previous studies have suggested that the FOXP3/IL-17 ratio may be a useful indicator for representing the severity of tissue injury, allograft dys-function and for predicting the clinical outcome of ATCMR [44]. However, the precise roles of Treg and Th17 cells in renal transplantation rejection need to be further explored. Morever, our study showed that there was no correlation between the number of CD4^+^CD25^+^Foxp3^+^ T cells and the number of CD4^+^IFN-γ^+^IL-17^−^ Th1 cells, which was inconsistent with previous research that showed suppression of Th1 cell-mediated responses by Tregs through inhibition of monocyte-derived IL-6 [30]. This possibility could be attributed to the difference in the internal circumstances of the patients. Previous studies have reported that multiple inflammatory cytokines played a key role in transplantation rejection response [31–33], such as Th1-type (IL-2, IFN-γ, TNF-α) and Th17-type (IL-17) cytokines. IL-2 is the critical mediator of acute rejection whereas IFN-γ can mediate separate functions at the same target organ during Graft-versus-host disease (GVHD) [30–32]. TNF-α is a lymphocyte and macrophage derived cytokine that is pleiotropic in its actions. Its proinflammatory function suggest that it may play an important role in initiating and orchestrating the rejection response. Studies demonstrating a correlation in the expression of TNF-alpha with the severity of the rejection episode have placed TNF-alpha as a prime candidate marker of transplantation rejection [51, 52]. IL10 is a potent synthesis inhibitory factor and anti-inflammatory, and is an important immunoregulatory component in the cytokine network of RTR’s [53]. IL-17 can induce the expression of proinflammatory TNF-α, and MCP-1 and MIP-1 to promote tissue inflammation [33]. In the present study, we found that RTR patients displayed higher levels of serum IL-2, IFN-γ, TNF-α and IL-17, but not IL-4 and IL-6, whereas TS patients displayed higher levels of serum IL-10 compared to pre-transplant status. More importantly, the serum levels of TNF-α and IL-17 were positively correlated with the concentrations of serum Cr, whereas IL-10 level was negatively correlated with the concentration of serum Cr in all the four post-transplant groups. These data indicated that an imbalance of serum Th1- and Th17-type cytokines may be a positive regulator during renal transplantation rejection process. Conceivably, the change in inflammatory cytokines may be a new target for designing immunotherapy for the intervention of renal transplantation rejection response.

## Conclusion

In summary, our data showed a significantly increased number of circulating Th1 and Th17 cells as well as higher levels of serum inflammatory cytokines in ESRF patients. RTR displayed lower number of Tregs, Treg to Th17 cells ratio and serum IL-10, and higher numbers of Th1 and Th17 and related cytokines compared to the pre-transplant and post-transplant TS patients. These novel finding suggested that effector CD4^+^ T cells may be controlled by Tregs during renal transplantation rejection and the imbalance of Treg/Th17 axis may be associated with the process of renal transplantation rejection. The limitations of this study include small sample size and lack of functional studies of different types of CD4^+^ T cells. Thus, further studies in larger population are warranted.
